# Investigation on the Air Permeability and Pore Structure of Concrete Subjected to Carbonation under Compressive Stress

**DOI:** 10.3390/ma15144775

**Published:** 2022-07-07

**Authors:** Cheng Zhang, Xinyu Shi, Ling Wang, Yan Yao

**Affiliations:** State Key Laboratory of Green Building Materials, China Building Materials Academy, Beijing 100024, China; zhangc0228@163.com (C.Z.); shixy19940426@126.com (X.S.); yy@cbmamail.com.cn (Y.Y.)

**Keywords:** concrete, carbonation, compressive stress, pore structure, gray relational analysis

## Abstract

Concrete structures have to withstand the combined effects of external load and environmental factors. Therefore, it is meaningful to study the durability of concrete under compression and carbonation. The air permeability coefficient (*k_Au_*) and pore structure of concrete under uniaxial compression and carbonation were measured by the Autoclam method and mercury intrusion porosimetry (MIP). The Autoclam test results showed that the concrete *k_Au_* changed in a concave parabolic manner with the compressive stress level, and the inflection point of the stress level was 45%. The MIP results showed that the characteristic pore structural parameters (porosity, average pore diameter, median pore diameter by area, and median pore diameter by volume) first decreased and then increased with the stress level change. The change in concrete microstructure was a result of the combined effect of pore filling, decalcification, and densification, as well as the split effect. The key pore structural parameters affecting *k_Au_* were confirmed using gray relational analysis (GRA). The top three parameters with the highest correlation with the carbonated concrete *k_Au_* were porosity (gray relational grade *γ_i_* = 0.789), median pore diameter by volume (*γ_i_* = 0.763), and proportion of transition pore volume (*γ_i_* = 0.827). Furthermore, the regression analysis showed a good linear relation between *k_Au_* and the important pore structural parameters.

## 1. Introduction

Carbonation, a chemical reaction between the CO_2_ in air and hydration products in cement pastes, is attracting more attention from scholars. The main reason for focusing on this topic is that concrete carbonation reduces the pH of the pore solution and then destroys the passivation film protecting the reinforcement in the concrete structure. Furthermore, carbonation causes serious damage to concrete structures because the CO_2_ concentration in the air has increased considerably in recent years. Therefore, it can be estimated that damage to concrete structures due to CO_2_ attacks will become more serious.

**Figure 1:**
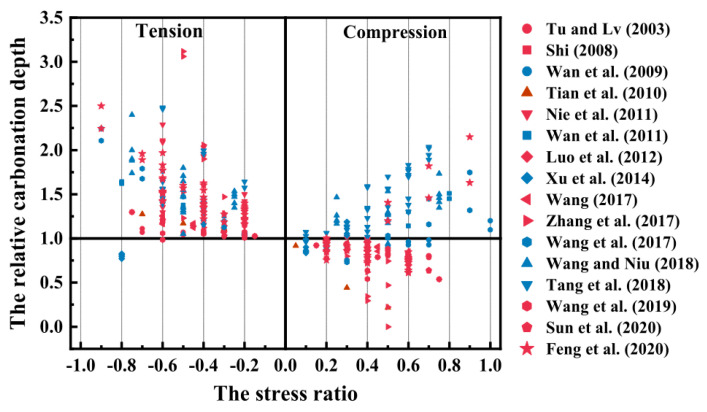
The effect of tension and compression on the carbonation depth from the annotated bibliography [29,30].

Many factors affect the concrete carbonation: water–cement ratio [1,2], supplementary cementitious materials [1,3,4,5,6,7,8], CO_2_ concentration [9,10,11], relative humidity [9,12,13,14], and applied load [8,15,16,17,18,19,20,21,22,23,24,25]. Much work based on the standard test methods and specifications has been carried out on concrete specimens in a load-free status without considering the effect of applied load. However, in actual structures, concrete carbonation occurs while it bears the external load. A series of attempts have been made to study concrete carbonation damage under stress. Wan et al. [18] and Tang et al. [19] reported that the carbonation rate rises and then falls with compressive stress. Han et al. [20] found that tensile stress deepened the carbonation depth, and compressive stress restrained concrete carbonation. They also noted a linear relationship between the tensile stress and the carbonation depth. Zeng and Wang et al. [26] and Zeng et al. [27] investigated the effects of different stress ratios on the diffusivity of porous cementitious materials and found that uniaxial loads decrease pore areas but increase the lengths of the pore channels for mass diffusion, which eventually causes a decrease in the effective diffusivity. Tang et al. [15], Lei et al. [16], and Wang et al. [28] presented matrix generated cracks that led to a larger CO_2_ diffusion rate and deeper carbonation depth under high stress. As shown in Figure 1, the recently published annotated bibliography [29,30] by RILEM TC 281-CCC WG4 collected the carbonation test of concrete under stress published over 20 years. It can be seen that tensile stress facilitates carbonation while compressive stress has a complicated relationship with carbonation depth. Experimental results showed that the synergetic effects of mechanical load and environmental actions on concrete durability should not be neglected, as Yao pointed out [21,22,30].

The porosity, interconnectivity of pores, and microcracks of concrete are the most important microstructure parameters that critically influence gas transport [31,32,33,34]. Furthermore, concrete is subjected to the external actions of stress, chloride, freezing–thawing, carbonation, and sulfate, which can change the pore structure. Therefore, we can surmise that the test gas permeability coefficient also will change.

The gas permeability coefficient is evaluated for concrete carbonation damage, and some studies were based on experiments using concrete specimens in a load-free state. Dhir et al. [35] and Torrent et al. [36,37] proposed a strong linear relationship between the air permeability coefficient and carbonation rate, whereas Salvoldi et al. [38] provided a prediction model of carbonation depth development based on the oxygen permeability coefficient.

Furthermore, some scholars studied gas permeability under stress. Yang et al. [39] studied the splitting load with stress levels between 50% and 80% for the air permeability of UHPC and found that the gas permeability of UHPC increased with the splitting load-induced residual crack-opening displacement. Djerbi Tegguer et al. [40] found that the gas permeability was more sensitive to concrete damage by uniaxial compression than chloride diffusion and increased with residual strain. Zhou et al. [41] showed a clear correlation between gas permeability and concrete damage under the cyclic load.

Even though concrete is subjected to the synergetic action of stress and environmental actions, it is not easy to achieve axial compression and carbonation on specimens simultaneously in labs. Fortunately, RILEM TC 246-TDC [22,42] provided a test rig to stabilize the stress and real-time monitoring. Therefore, we can put the loaded specimen together with the test rig in a carbonation chamber to create the synergetic test of the concrete under compression and carbonation. Tang et al. [19] first explored the gas permeability coefficient of concrete under compression with stress levels of 0%, 30%, and 60%. However, the relationship between the air permeability coefficient and the concrete pore structure under carbonation and compression has not been clarified. 

In this paper, a systematic analysis was performed to investigate the simultaneous influence of carbonation and compression (C–C) stress on air permeability and pore structure of concrete, during which time the carbonation depth (*D_c_*), air permeability coefficient (*k_Au_*), and characteristic pore structural parameters were tested. The gray relational analysis (GRA) method was used to analyze the relation between *k_Au_* and the pore structure of concrete under C–C, and, from this, a model between *k_Au_* and the key pore structural parameters was developed. This research will be helpful for improving the understanding of concrete deterioration under C–C.

## 2. Materials and Methods

### 2.1. Raw Materials

Type I ordinary Portland cement (OPC) was used, and its chemical and physical properties are shown in Table 1 and Table 2. River sand with an apparent density and fineness modulus of 2680 kg·m^−3^ and 2.80, respectively, was used as fine aggregate. Crushed limestone prepared from 5–10 mm particles and 10–20 mm particles at a weight ratio of 4:6 was used as coarse aggregate. The other components were tap water and polycarboxylate superplasticizers with a solid content of 29%.

### 2.2. Mix Proportion and Concrete Specimens

The concrete mix proportion is shown in Table 3, and the slump and prism compressive strength are listed in Table 3.

Prism specimens with dimensions of 100 × 100 × 300 mm were prepared for the test. Table 4 presents the detailed information of the specimens. 

### 2.3. Test Devices

#### 2.3.1. Compression Test Rigs

**Figure 2:**
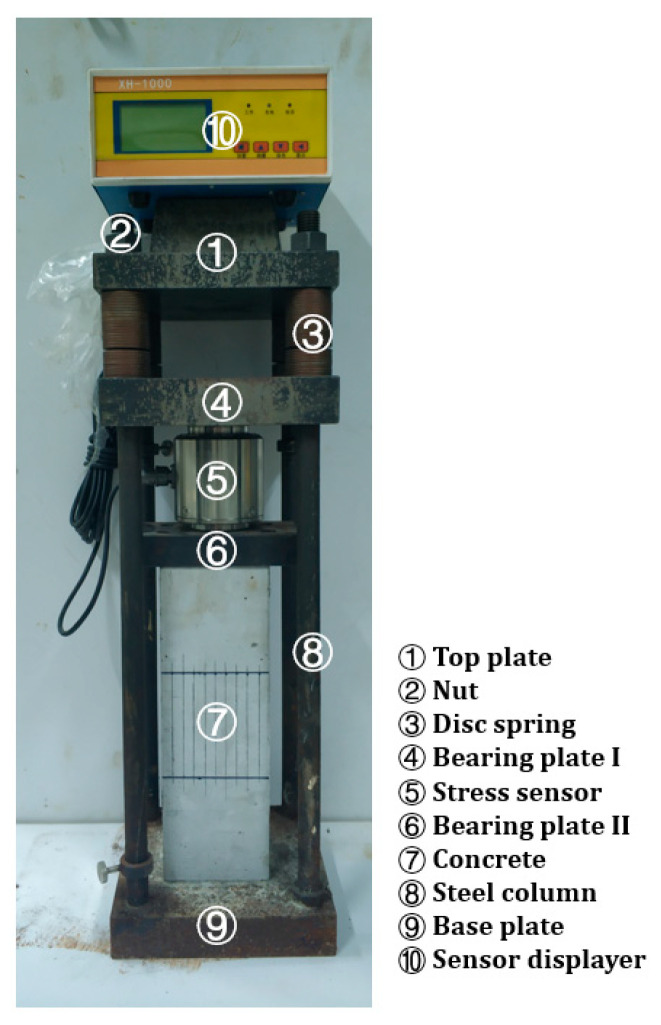
Experimental setup for compressive tests.

The compression test rigs used in the experiment, as shown in Figure 2, were consistent with the RILEM (International Union of Laboratories and Experts in Construction Materials, Systems, and Structures) recommendation [22,42]. Stress sensors with a precision of 0.01 kN were used to monitor the applied loads throughout. Compensation was needed to ensure a constant stress if it fell by 10%. Depending on the stress level, compensation was usually conducted once every 3–7 days. 

#### 2.3.2. Autoclam Test System

**Figure 3:**
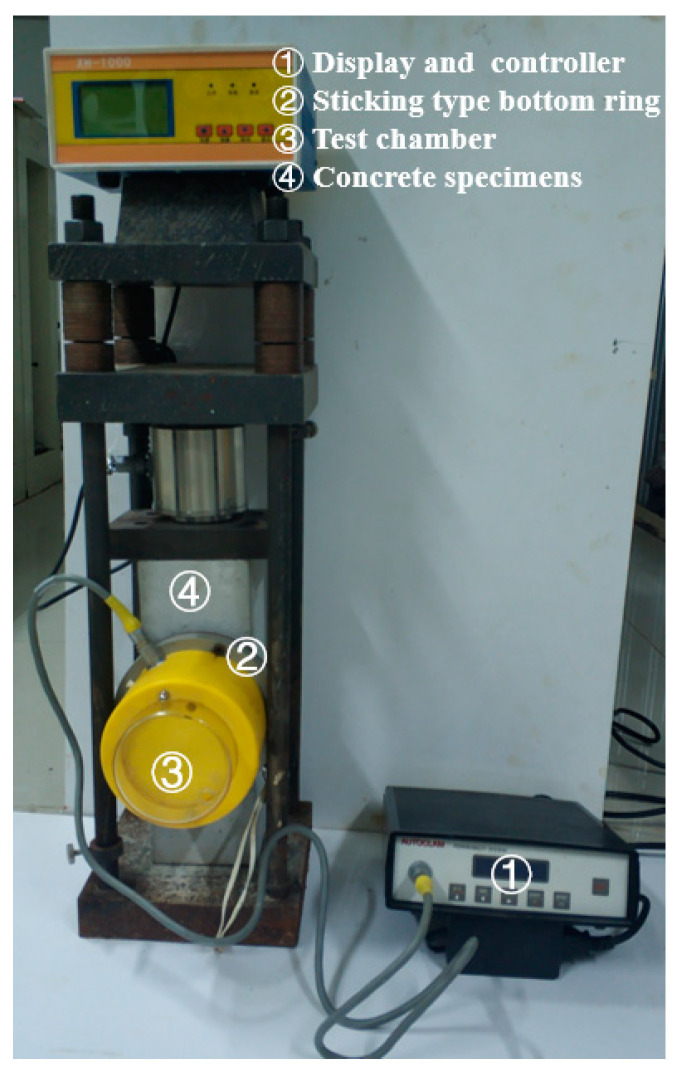
Autoclam test system.

The gas permeability coefficient of concrete was tested using the Autoclam test system [43,44,45], as shown in Figure 3.

The bonded bottom ring (②) was fixed to the exposed surface of the concrete specimen (④) using a thermosetting adhesive; then air pressure of 0.5 bar was applied to the test chamber (③) using a syringe. The air in the chamber gradually penetrated into the concrete, and the display (①) showed the attenuation of test-chamber pressure in real time. Each test took 15 min, and the system recorded the air pressure every minute. The natural logarithms of the air pressure were plotted against time to calculate the air permeability index (API). The slope of the last 10 data points was reported as the API (Ln(bar)/min). The air permeability coefficient of concrete (m^2^) of concrete was calculated according to Equation (1).

(1) $$k_{Au}=8.395\times 10^{-16}{\left(API\right)}^{0.8754}.$$

#### 2.3.3. Mercury Injection Apparatus

The pore structural parameters—porosity, average pore diameter (*d_A_*), median pore diameter by area (*d_M-A_*), median pore diameter by volume (*d_M-V_*), and pore volume distribution (PVD)—were measured by mercury intrusion porosimetry (MIP) through tracking pressure and the intrusion volume of mercury using the AutoPore IV 9510 (Micromeritics Instruments Corporation, Norcross, GA, USA). The parameters had a maximum intrusion pressure of 33,000 psi, meaning that the mercury could intrude into the pore diameter between 0.006 μm and 1000 μm.

### 2.4. Test Procedure

**Figure 4:**
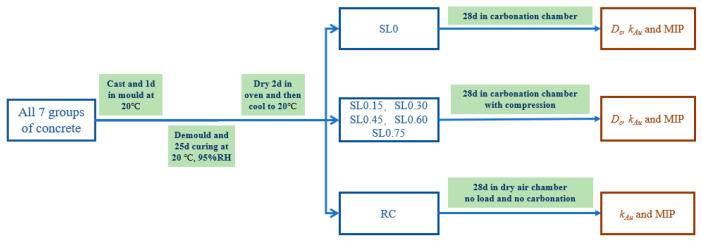
Test procedure and test items.

The test procedure is shown in Figure 4. 

All the concrete specimens were demolded for 1 day and placed in a standard curing room at 20 ± 2 °C and 95% relative humidity (RH) for 25 days. Then, all specimens were put into an electric drying oven at 60 °C for 2 days to evaporate excess water and balance the relative humidity within the specimens. Finally, they were taken out and cooled down to room temperature (20 °C) before the subsequent tests were carried out. The experiments were carried out according to Chinese standard GB/T 50082-2009.

#### 2.4.1. Accelerated Carbonation and Compression

To meet the needs of practical engineering, this study chose a wider range of stress levels: SL0, SL0.15, SL0.30, SL0.45, SL0.60, and SL 0.75. Six groups of concrete specimens (SL0, SL0.15, SL0.30, SL0.45, SL0.60, and SL0.75; three specimens for each group) were tested in the following steps:

1. To prevent carbonation damage that may cause eccentric compression, the concrete specimens were sealed with aluminum foil, leaving two opposite sides for carbonation.

2. The wrapped specimens were properly placed on the test rig as shown in Figure 2. One concrete specimen was placed on each rig. Compressive loads of six stress levels were applied to the specimens: 0%, 15%, 30%, 45%, 60%, and 75% of the ultimate compressive strength of the concrete’s *f_c_* in Table 3.

3. The loaded specimen and the test rig were put into a carbonation chamber at 20 ± 3 °C, 65 ± 5% relative humidity, and 20 ± 3% CO_2_ concentration of for 28 days of accelerated carbonation.

4. After the 28 days of accelerated carbonation and compression, the *k_Au_* of carbonated concrete was measured according to the test method in Section 2.3.2 before the specimen was unloaded.

5. The specimen was unloaded after *k_Au_* measurement. Then, the carbonation depth and pore structure of the carbonated concrete were measured on the unloaded specimens.

① Carbonation depth (*D_C_*)

The carbonated concrete specimens were split from the middle using an electric universal testing machine. The freshly exposed surface was painted with 1% phenolphthalein alcohol solution. The uncarbonized region turned red due to the high pH, and the area where the carbonization reaction occurred turned grey due to the decreased pH. The thickness of the gray zone was measured as the carbonation depth, which was measured every 10 mm along each split surface (18 measuring points for each specimen). The average of these values was taken as the carbonation depth of the specimen at the corresponding stress level. The *D_C_* of each specimen was measured using a vernier caliper with an accuracy of 0.1 mm.

② Pore structural parameters

The carbonated concrete specimen (three specimens for each group) was broken into smaller pieces, and then 3–5 mm particles were selected from the cement mortar fragment in the fully carbonated zone. Next, the samples were immersed in alcohol to terminate cement hydration for 2 days. Then, the samples were dried at 60 °C in an oven for 2 days to remove alcohol and evaporate water. Finally, the porosity (*d_A_*, *d_M-A_*, and *d_M-V_*) and the PVD of the carbonated concrete were measured using the MIP method in Section 2.3.3.

#### 2.4.2. Comparison Test without Carbonation and Compression

The reference concrete (RC) specimen (three specimens per group) was tested in the following steps:

1. The specimens were stored in the dry air chamber at 20 ± 3 °C and 65 ± 5% relative humidity for 28 days. This operation aimed to keep the RC specimen at the same relative humidity as the groups undergoing accelerated carbonation in Section 2.4.1.

2. The *k_Au_* and pore structure parameters of the RC specimen were tested at the same age as SL0. The test methods are described in Section 2.3.2 and Section 2.3.3.

## 3. Test Results and Discussion

### 3.1. Air Permeability Coefficient and Carbonation Depth of Concrete

**Figure 5:**
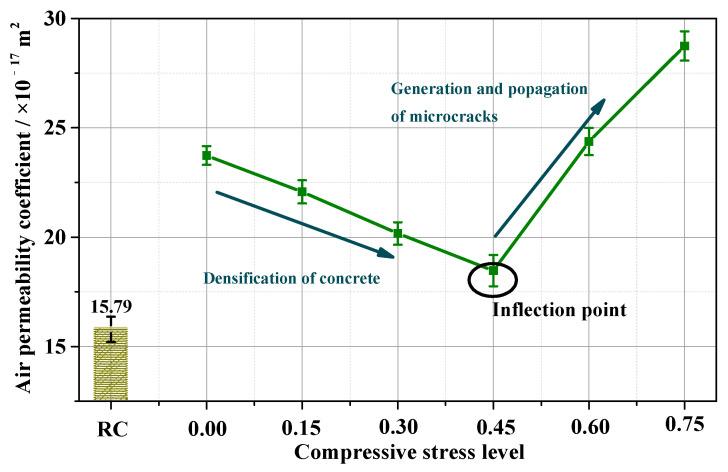
Air permeability coefficient of concrete under different stress levels.

The *k_Au_* of concrete under C–C is shown in Figure 5. In addition, the *k_Au_* of the RC specimen (without carbonation and compression) is plotted in Figure 5 as a yellow column. 

Compared with the *k_Au_* of RC (15.79 × 10^−17^ m^2^), the *k_Au_* of SL0 (with carbonation only) increased by 50.28%. Meanwhile, the *k_Au_* of SL0.15, SL0.30, SL0.45, SL0.60, and SL0.75 (with the combination of compression and carbonation) increased by 40%, 28%, 17%, 54%, and 82%, respectively. The increased tendencies of the *k_Au_* illustrate that carbonation and the combination of carbonation and compression both worsened gas permeability resistance.

**Figure 6:**
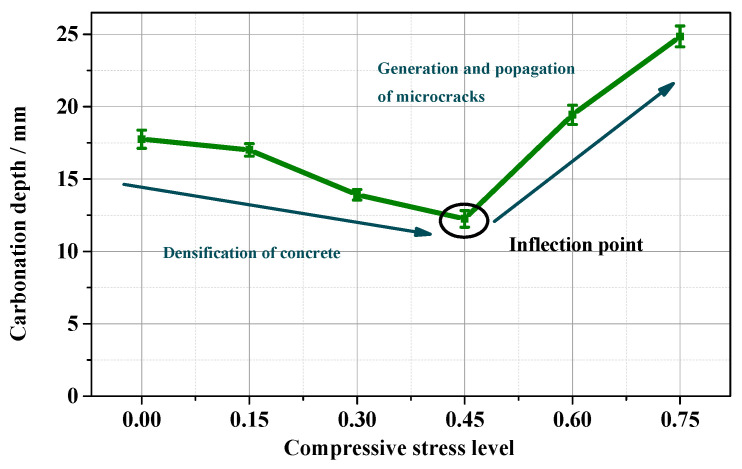
Carbonation depth of concrete under different compressive stress levels.

As shown in Figure 5, the *k_Au_* of concrete under C–C initially decreased with the stress level, but then began to increase rapidly after the compressive stress level exceeded 0.45. This result was consistent with the reports from Tang et al. [19] and Banthia et al. [32]. The *k_Au_* of concrete had a minimum value at the 0.45 stress level. We noticed that the minimum concrete carbonation depth was at the stress ratio of 0.45 (Figure 6). Researchers also reported that the minimum chloride diffusion coefficient *D_C_* under a compressive load [17,21,42] was obtained at the stress level of 0.4–0.6. The occurrence of an inflection point on the *k_Au_* curve with stress levels resulted from two effects acting together as *D_C_*. Compression and carbonation, on the one hand, caused the concrete microstructure to densify [1] but, on the other hand, caused microcracks to propagate [24].

The *k_Au_* of concrete is a good indicator for reflecting the ability of concrete to resist the penetration of harmful substances, such as CO_2_, sulfate ions, and chloride ions. For example, the *k_Au_* values in Figure 5 show that the compression improved air permeability resistance when a lower compressive stress (<0.45) was applied. On the other hand, the air permeability resistance of concrete was seriously worsened after a stress > 0.45 was applied.

### 3.2. The Change in Pore Structure of Concrete

#### 3.2.1. Accelerated Carbonation and Compression

**Figure 7:**
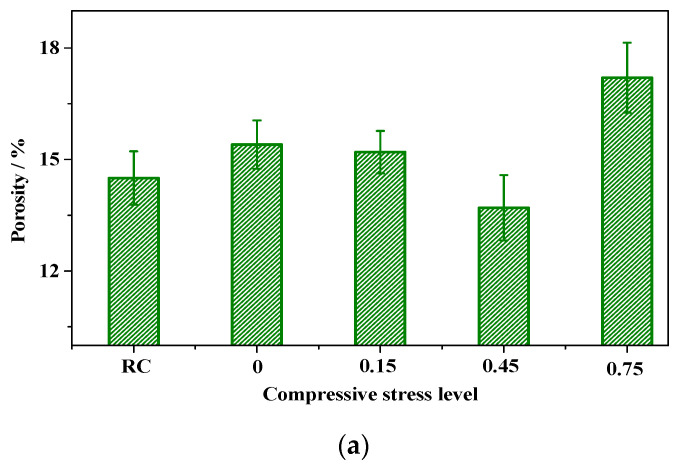

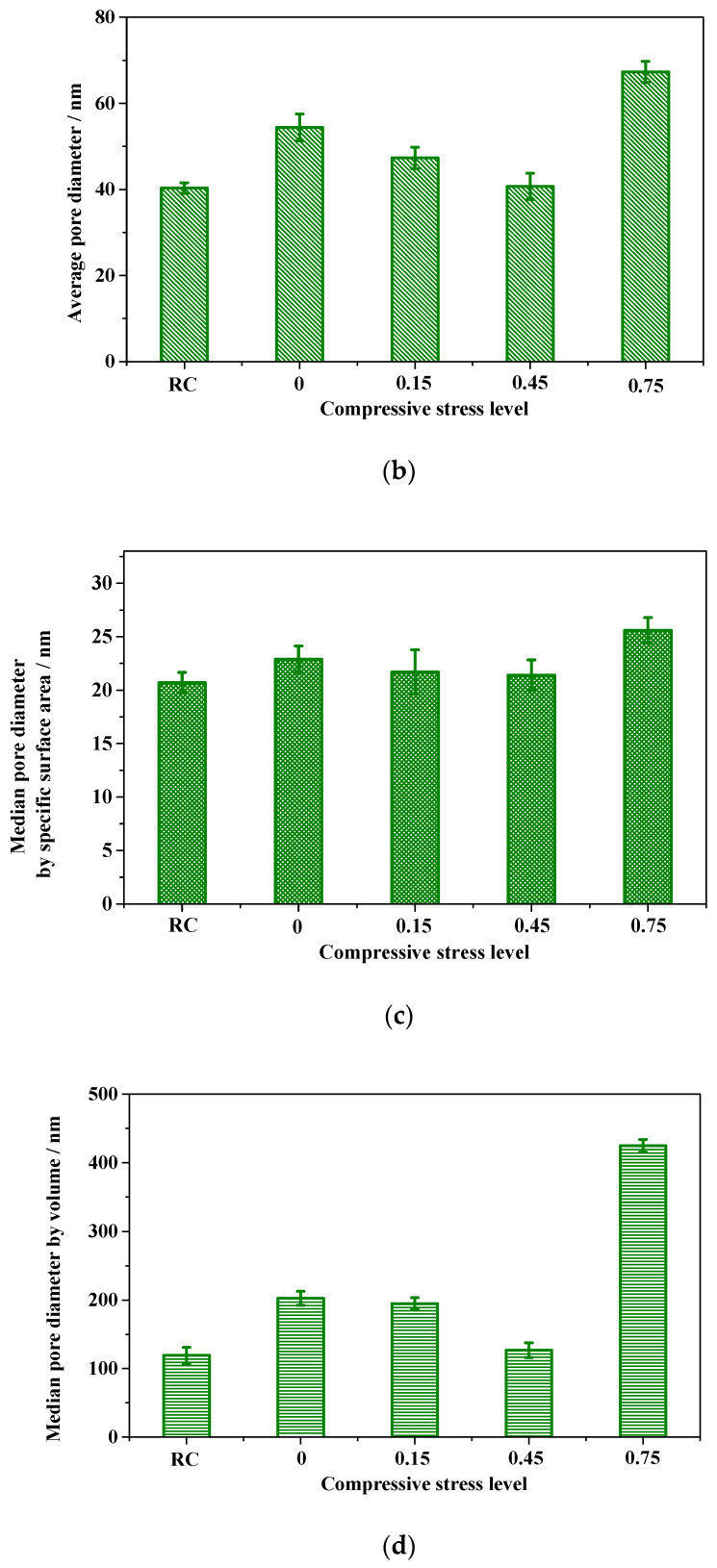
Characteristic pore structural parameters of concrete: (**a**) porosity; (**b**) average pore diameter; (**c**) median pore diameter by area; (**d**) median pore diameter by volume.

MIP was used to measure the pore microstructure at four stress levels. The characteristic parameters of pore structure under carbonation and compression—porosity, average pore diameter *d_A_*, median pore diameter (area) *d_M-A_*, and median pore diameter (volume) *d_M-V_*—are listed in Figure 7, as well as the characteristic pore structure parameters of the RC without carbonation and compression.

From Figure 7, we can find that the porosity, *d_A_*, *d_M-A_*, and *d_M-V_* of concrete SL0 increased by 6% (Figure 7a), 35% (Figure 7b), 11% (Figure 7c), and 70% (Figure 7d) compared to that of the RC, respectively. The increase in the four parameters indicated that carbonation lowered compactness. It should be noted that these results are different from those in the literature [46,47,48], in which the four parameters decreased after carbonation. A possible reason is that the concrete had a higher water–cement ratio (0.6) and a higher CO_2_ concentration (20%) than in our carbonation test. The carbonation of C–S–H at a higher CO_2_ concentration increased porosity and pore diameters [1,47,49]. 

Comparing the characteristic pore structural parameters of SL0.15, SL0.45, and SL0.75 with that of SL0, we found the influence of compression on concrete microstructure. The porosity of carbonated concrete under compressive stress decreased by 1.3% (SL0.15), 11.04% (SL0.45), and −11.69% (SL0.75) compared to that of SL0, as shown in Figure 7a. The porosity of concrete under compression and carbonation decreased slightly and then increased rapidly with the stress levels. The porosity decrease in the low stress level indicated that the compactness increased. The compactness decreased at the high stress level because higher stress promotes pore-wall [50] deterioration and accelerates pore destruction.

As presented in Figure 7b–d, the change tendencies of *d_A_*, *d_M-A_*, and *d_M-V_* with stress levels all showed an increasing trend after a short reduction. When the stress level was less than 0.45, the *d_A_*, *d_M-A_*, and *d_M-V_* decreased with the stress level, suggesting that the concrete matrix became more and more compact with the increase in stress. The decreased tendency of *d_A_*, *d_M-A_*, and *d_M-V_* at lower stress levels indicated that the concrete pores may be greatly compressed under a small external compressive load. Thus, the compression of gradually compacting concrete and reduction in the transition of harmful ions is defined as “the densification effect”. 

When the stress level exceeded 0.45, the *d_A_*, *d_M-A_*, and *d_M-V_* increased with the stress level. These results implied that compression and a stress level > 0.45 worsened the microstructure of concrete and damaged pore walls. We defined this worsening effect as “the split effect”.

#### 3.2.2. Pore Volume Distribution (PVD)

Concrete is a porous, nonhomogeneous material; hence, its internal pore distribution is tortuous and complicated. According to Houst and Wittmann [51], the diffusion of gas in concrete is related to the mean free path *λ* of gas (calculated by Equation (2)) and the pore size. The diffusion of gas in concrete can be divided into three different types:

(1) Fick diffusion or normal molecular diffusion frequently occurs in pores with a diameter larger than 10*λ*.

(2) Knudsen diffusion refers to the phenomenon of gas molecules bouncing back from the pore’s wall when the size of the gas molecule is close to that of the pore size. The collision between the molecules can then be ignored [52]. Knudsen diffusion usually occurs in pores with a diameter smaller than *λ*.

(3) Transition diffusion, unlike Fick and Knudsen diffusion, usually occurs in pores with a diameter between *λ* and 10*λ*.

According to Peter et al. [53] and Muntean et al. [54], a pore’s saturation degree (water filling of the pores, calculated by Equation (3)) also greatly influences the air diffusion. A higher saturation degree results in slower air diffusion. Therefore, gas diffusion in saturated pores could be almost ignored.

In this paper, we used air as a transmission medium to measure the *k_Au_* of concrete, and the temperature and relative humidity for carbonation were 20 ± 3 °C and 65 ± 5%, respectively. The mean free path of air λ was calculated as 69 nm according to Equation (2) [51].

(2) $$\lambda =\frac{R_{gas}T}{N\pi d^{2}P},$$

where λ is the mean free path of air in nm, $R_{gas}$ is the gas constant (8.3143 J/(K·mol)), T is the temperature (293 K), N is Avogadro’s number (6.022 × 10^23^), d is the molecular diameter of air (3.5 × 10^−10^ m), and P is the pressure (101,325 Pa).

The saturated pore diameter was calculated as 10 nm according to Equation (3) [53,54].

(3) $$d_{sp}=\frac{2\gamma V_{m}}{R_{gas}T\ln\left(\mathrm{RH}/100\right)}+0.425{\left[-\log\left(\mathrm{RH}/100\right)\right]}^{-0.31},$$

where γ is the surface tension of water (72.8 mN/m), $V_{m}$ is the molar volume of water molecules (18.016 mL), $R_{gas}$ is the gas constant (8.3143 J/(K·mol)), T is the temperature (293 K), and RH is the relative humidity (65%).

We divided the tested concrete pores into four levels according to pore diameter on the basis of these two calculations. Fick pores had a diameter larger than 690 mm, while the transition pores had a diameter between 690 and 69 nm. Knudsen pores had a diameter between 69 and 10 nm, while the saturated pores had a diameter between 69 and 10 nm.

**Figure 8:**
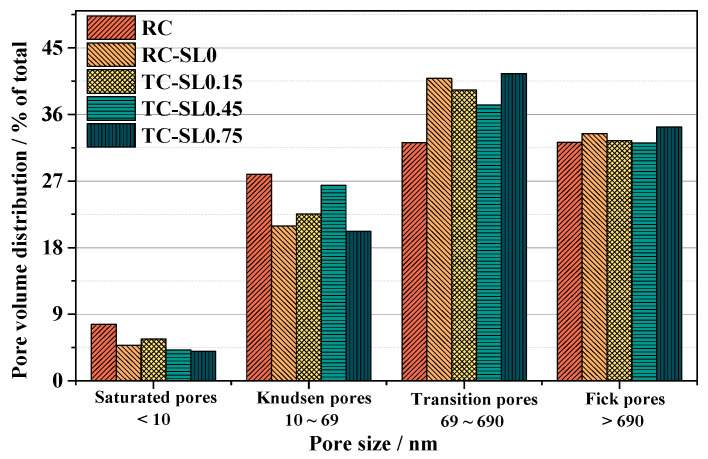
PVD of the concrete under C–C using different CO_2_ diffusion approaches.

The PVD of concrete under C–C according to these four pore ranges is illustrated in Figure 8. The change values of the PVD of concrete under C–C compared with those of RC and SL0 were calculated, and the results are shown in Table 5.

The mixing proportions of the RC and SL0 were the same. However, after 28 days of carbonation, the proportion of Knudsen pores (PROP_K_) of SL0 was smaller than that of the RC, and the proportion of transition pores (PROP_T_) of SL0 was larger than that of the RC.

Compared with concrete SL0, the increment in TC’s PROP_K_ increased early and then decreased with stress level; the increment in TC’s PROP_T_ first decreased and then increased with stress levels, as shown in Figure 8 and Table 3. The results indicated that the carbonation reaction increased the PROP_T_, directly increasing the *k_Au_*.

It was noticed that the proportion of the saturated (PROP_S_) and Fick pores (PROP_F_) of SL0 barely changed compared to RC, indicating that the carbonation reaction did not affect the smaller (<10 nm) or larger pores (>690 nm). Compared with concrete SL0, Figure 8 and Table 5 show that the PROP_S_ and PROP_F_ of concrete under C–C were almost unchanged as stress levels increased. The maximum variation values were 0.89% and −1.26% for PROP_S_ and PROP_F_, respectively. Therefore, we focused on the PROP_T_ and PROP_K_ of concrete under the carbonation and compression.

### 3.3. The Degradation of Concrete under Carbonation and Compression

An analysis of concrete degradation under carbonation and compression needs to consider its effects on the microstructure.

#### 3.3.1. Carbonation Action

Carbonation results in the neutralization reaction of the cement hydrates, including calcium hydroxide (CH), calcium silicate hydrates (C–S–H), and ettringite.

CH and C–S–H are the most abundant cement hydration products and the earliest to react with carbon dioxide. The neutralization reactions of CH and C–S–H and the solid-phase volume changes are shown in Equations (4) and (5) [55].

(4) $$\begin{array}{c} Ca{\left(OH\right)}_{2}\left(s\right)+CO_{2}\left(aq\right)\to CaCO_{3}\left(s\right)+H_{2}O\left(l\right).\\ \mathrm{Volume}\;\mathrm{change}:\;\mathrm{CH},\;1\;{\mathrm{cm}}^{3};\;{\mathrm{CaCO}}_{3},\;1.12\;{\mathrm{cm}}^{3}. \end{array}$$

(5) $$\begin{array}{c} Ca_{9}H_{2}Si_{6}O_{18}{\left(OH\right)}_{8}\cdot 6H_{2}O\left(gel\right)+9CO_{2}\left(aq\right)\\ \;\to 9CaCO_{3}\left(s\right)+6SiO_{2}\left(gel\right)+11H_{2}O\left(l\right).\\ \mathrm{Volume}\;\mathrm{change}:\;\mathrm{CSH},\;1\;{\mathrm{cm}}^{3};\;{\mathrm{CaCO}}_{3},\;1.38\;{\mathrm{cm}}^{3}. \end{array}$$

Equations (4) and (5) indicate that the carbonation reactions cause the precipitation of calcium carbonate (CaCO_3_). The volume of CaCO_3_ produced by the carbonation reaction of CH and C–S–H increases by 12% [56] and 38% [57], respectively. During carbonation, the CH dissolves and leaves space for bigger pores, and the carbonation products develop inside them [58]. This clogging of the pores is called “the pore-filling effect”.

In addition, the C–S–H reacts with CO_2_ in a cascade of reactions that progressively removes Ca and water from the C–S–H gel via a sequence of C–S–H phases having a progressively lower Ca/Si ratio (Equation (6)), with the ultimate products being CaCO_3_(s) and SiO_2_ gel (Equation (7)) [55]. With increasing C–S–H carbonation, the Ca/Si ratio decrease causes concrete shrinkage, leading to the coarsening of the pore structure [1,47,55,56]. The pore coarsening caused by the carbonation of C–S–H is defined as “the decalcification effect”. It increases with a higher CO_2_ concentration and smaller Ca/Si ratio, showing a maximum at high-to-moderate relative humidity [1].

(6) $$\begin{array}{l} 2Ca_{9}H_{2}Si_{6}O_{18}{\left(OH\right)}_{8}\cdot 6H_{2}O\left(gel\right)+8CO_{2}\left(aq\right)\\ \;\to 2Ca_{5}Si_{6}O_{16}{\left(OH\right)}_{2}\cdot 9.5H_{2}O\left(gel\right)+8CaCO_{3}\left(s\right)+4.5H_{2}O\left(l\right). \end{array}$$

(7) $$\begin{array}{l} Ca_{5}Si_{6}O_{16}{\left(OH\right)}_{2}\cdot 9.5H_{2}O\left(gel\right)+5CO_{2}\left(aq\right)\\ \;\to 5CaCO_{3}\left(s\right)+6SiO_{2}\left(gel\right)+4.5H_{2}O\left(l\right). \end{array}$$

These two reactions brought two main effects to the pore and the pore wall, which affected the microstructure of concrete. First, the pore-filling effect led to a larger *PROP_T_* of concrete SL0 compared to RC (Figure 8), and the decalcification effect led to the increasing porosity of SL concrete compared to RC (Figure 7a).

**Figure 9:**
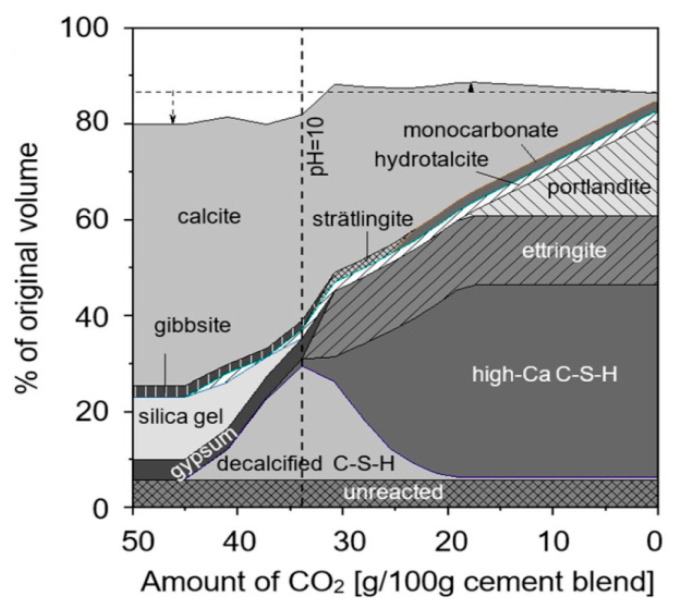
Thermodynamic modeling of the phase assemblage during carbonation of OPC concrete [1,59].

It is worth noting that these two effects were related to the exposure time or the amount of CO_2_ involved in the reaction. von Greve-Dierfeld et al. [1] and Shi et al. [59] revealed that the phase assemblage of Portland cement is a function of the amount of CO_2_ that reacted with the cement paste through a thermodynamic model (Figure 9). A greater CO_2_ content involved in the carbonation reaction, i.e., a longer exposure time, results in a more complete the carbonation of CH and C–S–H. 

#### 3.3.2. Compression Action

When the concrete was subjected to compression, the external mechanical load had two effects on the pore and the pore wall, which affected the microstructure. However, due to the different stress ratios applied, the internal pores and pore walls experienced positive and negative compression effects. When the concrete was subjected to a stress level less than 0.45, the concrete experienced the positive compression (densification) effect; conversely, when the concrete was subjected to a stress level larger than 0.45, it experienced the negative compression (split) effect.

#### 3.3.3. The Combined Actions of Carbonation and Compression

According to the above analysis, the main effects to the concrete microstructure under carbonation and compression are shown in Table 6.

The pore-filling effect clogs the pores and improves the permeability resistance of carbonated concrete [60]. Nevertheless, the decalcification effect coarsens the pores and reduce the permeability resistance [1]. However, concrete SL0 had a higher air permeability (Figure 5) than RC, indicating the decreased permeability resistance of carbonated concrete. Therefore, when the concrete was subjected only to carbonation., the decalcification effect was larger than the pore-filling effect.

When the concrete was subjected to compression, the air permeability decreased and then increased with the stress levels (see Figure 5); the *k_Au_*, *D_c_*, porosity, *d_A_*, *d_M-A_*, and *d_M-V_* of concrete SL0.45 were smaller than those of SL0, indicating that the cement matrix of SL0.45 became denser than the sample of SL0, implying that, in the densification, the pore-filling effects prevailed over the dual effects of densification decalcification.

The microstructure of concrete become looser and more porous, and many cracks appeared when the stress levels increased from 0.45 to 0.75. The higher external loads dominated the split of the pore walls inside the concrete, accelerating pore microstructure deterioration. Moreover, decalcification of C–S–H could further promote the destruction of the pore structure. The carbonation products filling the pores could not compensate for the shortcomings of the higher stress levels or the decalcification of C–S–H to generate microcracks that led to structure destruction. The dual effects of the split and decalcification effects prevailed over the split and pore-filling effects.

That is to say, when concrete was under the compression and carbonation, the degradation was dominated by the applied stress level. When it was below 0.45, the pores were compressed by the external compressive load and became filled with carbonation products, which made the concrete matrix denser and improved air permeability resistance. On the other hand, when stress levels went beyond 0.45, the pores were further compressed. Meanwhile, the decalcification of C–S–H in the pore walls prevailed over the filling effect of carbonation products, pore wall damage, and the decreasing air permeability resistance.

## 4. Correlation Analysis of Air Permeability Coefficient with Pore Structure Parameters of Concrete under Carbonation and Compressive Stress

### 4.1. The Gray Relational Analysis Method

Gray relational analysis (GRA) is a measurement method in gray system theory that analyzes uncertain relations between one main factor and others in a given system [61]. We used it to correlate the structural pore parameters with *k_Au_* to illustrate the changes induced by the carbonation and compression on the air permeability coefficient.

We took the *k_Au_* under C–C as the original reference sequence, represented by *X*(*l*). The eight specific pore structural parameters (porosity, *d_A_*, *d_M-A_*, *d_M-V_*, PROP_S_, PROP_K_, PROP_T_, and PROP_F_) were used as comparison sequences, represented by *Y_i_*(*l*). Data preprocessing was required since the range and unit in one data sequence may differ from those in others. The sequences were normalized on the basis of RC as follows:

(8) $$\{\begin{array}{c} X^{\prime }\left(l\right)=X\left(l\right)/X\left(1\right)\\ Y_{i}^{\prime }\left(l\right)=Y_{i}\left(l\right)/Y\left(1\right) \end{array},$$

where *l* = 1, 2, …, *m*, *m* is the number of experimental data items (*m* = 5); *i* = 1, 2, …, *n*, and *n* is the number of parameters (*n* = 8).

After data preprocessing, the gray relational coefficient $\gamma_{i}\left(l\right)$ for the *i-*th performance characteristics in the *l-*th experiment is expressed as follows [61,62,63]:

(9) $$\gamma_{i}\left(l\right)=\frac{{∆}_{min}+\xi \cdot {∆}_{max}}{{∆}_{i}\left(l\right)+\xi \cdot {∆}_{max}},$$

where ${∆}_{i}\left(l\right)$ is the absolute value of the difference between $X^{\prime }\left(l\right)$ and $Y_{i}^{\prime }\left(l\right)$, ${∆}_{min}$ is calculated by ${∆}_{min}=\begin{array}{c} min\\ \forall l \end{array}\begin{array}{c} min\\ \forall i \end{array}{∆}_{i}\left(l\right)$, ${∆}_{max}$ is calculated by ${∆}_{max}=\begin{array}{c} max\\ \forall l \end{array}\begin{array}{c} max\\ \forall i \end{array}{∆}_{i}\left(l\right)$, and ξ is the distinguishing coefficient, ξ∈[0,1]. We chose ξ = 0.5 according to [50,61,62,64].

We used the gray relational grade $\gamma_{i}$ as a numerical measure of the correlation between the reference and comparison sequences. The gray relational grade is expressed in Equation (10).

(10) $$\gamma_{i}=\frac{1}{n}\overset{n}{\underset{l=1}{\sum }}\gamma_{i}\left(l\right).$$

Closer sequences have a gray relational grade closer to 1.

### 4.2. Regression Analysis Based on the Gray Relational Analysis

The gray relational coefficients of concrete under C–C were calculated, and the results are presented in Table 7 and Table 8. The gray relational grades between *k_Au_* and the eight structural pore parameters are shown in Table 9. 

We found that the order of correlation between *k_Au_* and each of the structural pore parameters was PROP_T_ > porosity > *d_M-V_* > *d_M-A_* > *d_A_* > PROP_F_ > PROP_K_ > PROP_S_. That is to say, the porosity, *d_M-V_*, and PROP_T_ were the top three important parameters affecting the air permeability of concrete under C–C. Li et al. [41,65,66] proved that porosity and *d_M-V_* significantly influence the air permeability of concrete. The result of the gray relational grade showed that transition diffusion dominated the air diffusion in the carbonated OPC concrete, and the results indicated that the synergic effects of carbonation and compression significantly affected the pore diameter range of 69–690 nm. 

The relationship between the air permeability coefficient and the top three important pore structure parameters was established by regression analysis using Statistical Product and Service Solutions (SPSS) software [50], as shown in Equation (11). The regression coefficient (*R*^2^) reached 0.992, indicating a good linear relationship between the *k_Au_* and the three pore structure parameters.

(11) $$\frac{k_{Au}^{\prime }}{k_{Au}}=0.189\frac{p^{\prime }}{p}+0.181\frac{d_{M-V}^{\prime }}{d_{M-V}}+1.236\frac{PROP_{T}^{\prime }}{PROP_{T}}-0.617,$$

where $k_{Au}$ is the air permeability coefficient of concrete, p is the porosity of concrete, $d_{M-V}$ is the median pore diameter (volume) of concrete, $PORP_{T}$ is the volume proportion of transition pores (69–690 nm), $k_{Au}^{\prime }$ is the air permeability coefficient of damaged concrete after C–C, $p^{\prime }$ is the porosity of damaged concrete after C–C, $d_{M-V}^{\prime }$ is the median pore diameter (volume) of damaged concrete after C–C, and $PROP_{T}^{\prime }$ is the volume proportion of transition pores (69–690 nm) in damaged concrete after C–C.

The results demonstrated that the relationship between the air permeability coefficient and pore structural parameters of concrete subjected to C–C was linear. The *k_Au_* reflected the current state of the permeability resistance of concrete under C–C, and the *k_Au_* could be used to predict the service life of concrete subject to carbonation and compression on the basis of existing models [67].

## 5. Conclusions

This paper studied the deterioration of pore structure and air permeability of concrete under C–C. In addition, the correlation between pore structural parameters and *k_Au_* was investigated. The following conclusions were drawn:

(1) The *k_Au_* and the *D_C_* of concrete responded to stress levels showing concave parabolic trends. The minimum *k_Au_* and *D_C_* were obtained at the stress level of 0.45. The concrete deterioration deepened as the stress level exceeded 0.45. 

(2) When the applied stress levels of concrete were below 0.45, the dual effects of the densification by lower compression and the carbonation product filling of the pores were dominant under carbonation, promoting a compact concrete matrix. However, when the applied stress levels of concrete exceeded 0.45, the beneficial effect of pore filling was weakened, and the dual split effect of higher compression and decalcification of C–S–H accelerated structural deterioration.

(3) The GRA results showed that the top three parameters with the highest correlation to the carbonated concrete *k_Au_* were porosity, the proportion of the transition pore volume (PROP_T_), and the median pore diameter by volume (*d_M-V_*). These three parameters played an important role in the air permeability under C–C. Meanwhile, regression analysis showed a good linear relationship between air permeability and these pore structure parameters.

This work was a pilot study on the air permeability of concrete under carbonation and compression. The test of the air permeability coefficient and pore structural parameters was based on OPC concrete. However, supplementary cementitious materials (SCMs) are increasingly used in concrete, and their presence influences the hydrates formed in cementitious systems. Thus, more work is needed to understand the evolution of air permeability and its relationship with the pore structure in concrete under C–C.

## Figures and Tables

**Table 1:** Chemical compositions of cement.

Composition	SiO_2_	Al_2_O_3_	SO_3_	Fe_2_O_3_	MgO	CaO	LOI
Content (%)	22.11	4.43	2.62	3.13	2.28	62.38	2.04

**Table 2:** Physical properties of cement.

Density (kg·m^−3^)	Specific Surface (kg·m^−3^)	Setting Time (min)		Flexural Strength (MPa)		Compressive Strength (MPa)	
		Initial	Final	3 Days	28 Days	3 Days	28 Days
3127	355	99	159	5.8	8.6	28.1	51.2

**Table 3:** Mix proportion and mechanical properties of concrete.

Portland Cement (kg·m^−3^)	Fine Aggregate (kg·m^−3^)	Coarse Aggregate (kg·m^−3^)	Water (kg·m^−3^)	Superplasticizer (kg·m^−3^)	w/c	Slump (cm)	28 Day *f_c_* (MPa)
330	719	1162	198	0.7	0.6	11	34.93

**Table 4:** Detailed information of test specimens.

No.	Remarks	Test Items
RC	Reference concrete 1: no carbonation, no compression	*k_Au_*, MIP
SL0	Reference concrete 2: carbonation, no compression	*D_c_, k_Au_*, MIP
SL0.15	Test concrete: C–C (concrete under carbonation and compressive stress), stress level of 0.15	*D_c_, k_Au_*, MIP
SL0.30	Test concrete: C–C, stress level of 0.30	*D_c_, k_Au_*
SL0.45	Test concrete: C–C, stress level of 0.45	*D_c_, k_Au_*, MIP
SL0.60	Test concrete: C–C, stress level of 0.60	*D_c_, k_Au_*
SL0.75	Test concrete: C–C, stress level of 0.75	*D_c_, k_Au_*, MIP

**Table 5:** The PVD variation of concrete after C–C.

Conditions		Increment of PROP_s_	Increment of PROP_K_	Increment of PROP_T_	Increment of PROP_F_
SL0	ref. RC	−1.86	−6.98	+8.66	+1.18
SL0.15	ref. SL0	+0.89	+1.62	−1.54	−0.97
SL0.45	ref. SL0	−0.62	+5.48	−3.6	−1.26
SL0.75	ref. SL0	−0.81	−0.74	+0.66	+0.89

**Table 6:** Main effects affecting the microstructure of concrete under carbonation and compression.

	Compressive Stress Level			
	0	0–0.45	0.45	0.45–0.75
Pore-filling effect	++	++	++	++
Decalcification effect	++++	++++	++++	++++
Densification effect	/	+++++	+++++	/
Split effect	/	/	/	+++++

Note: + represents an effect (the more +, the greater the effect); / represents no correlation.

**Table 7:** Normalized data determined using Equation (8).

	*k_Au_*	Porosity	*d_A_*	*d_M-A_*	*d_M-V_*	PROP_S_	PROP_K_	PROP_T_	PROP_F_
RC	1	1	1	1	1	1	1	1	1
SL0	1.507	1.184	1.058	1.106	1.696	0.625	0.750	1.269	1.037
SL0.15	1.402	1.153	0.988	1.048	1.582	0.742	0.808	1.221	1.007
SL0.45	1.173	1.077	0.975	1.033	1.062	0.545	0.946	1.157	0.998
SL0.75	1.825	1.323	1.328	1.236	3.355	0.520	0.723	1.289	1.064

**Table 8:** The gray relational coefficient determined using Equation (9).

	Porosity	*d_A_*	*d_M-A_*	*d_M-V_*	PROP_S_	PROP_K_	PROP_T_	PROP_F_
RC	1	1	1	1	1	1	1	1
SL0	0.703	0.629	0.656	0.802	0.464	0.502	0.762	0.619
SL0.15	0.754	0.654	0.684	0.809	0.537	0.562	0.808	0.659
SL0.45	0.888	0.793	0.846	0.872	0.548	0.771	0.979	0.813
SL0.75	0.603	0.606	0.565	0.333	0.369	0.409	0.587	0.501

**Table 9:** Gray relational grade between *k_Au_* and the eight structural pore parameters under C–C.

Porosity	*d_A_*	*d_M-A_*	*d_M-V_*	Pore Volume Distribution			
				PROP_S_	PROP_K_	PROP_T_	PROP_F_
0.789	0.736	0.749	0.763	0.583	0.649	0.827	0.718

## Data Availability

Not applicable.

## Abbreviations

C–C	Carbonation and compressive stress	*k_Au_*	Air permeability coefficient
RC	Reference concrete	*D_C_*	Carbonation depth
SL	Stress level	*d_A_*	Average pore diameter
MIP	Mercury intrusion porosimetry	*d_M-A_*	Median pore diameter (area)
GRA	Gray relational analysis	*d_M-V_*	Median pore diameter (volume)
PVD	Pore volume distribution	PROP_S_	Proportion of saturation pores
C–S–H	Calcium silicate hydrates	PROP_T_	Proportion of transition pores
CH	Calcium hydroxide	PROP_K_	Proportion of Knudsen pores
SPSS	Statistical Product and Service Solutions software	PROP_F_	Proportion of Fick pores
		*γ*	Gray relational grade
